# Commentary: Polygenic risk for breast cancer: in search for potential clinical utility

**DOI:** 10.1093/ije/dyab230

**Published:** 2021-10-31

**Authors:** Tingting Wang, Mika Ala-Korpela

**Affiliations:** 1 Baker Heart and Diabetes Institute, Melbourne, VIC, Australia; 2 Computational Medicine, Faculty of Medicine, University of Oulu and Biocenter Oulu, Oulu, Finland; 3 Center for Life Course Health Research, University of Oulu, Oulu, Finland; 4 NMR Metabolomics Laboratory, School of Pharmacy, University of Eastern Finland, Kuopio, Finland

The use of a polygenic risk score (PRS) as an independent risk factor for common diseases is becoming mainstream.^1–3^ The initial hype on their clinical applicability appears to be maturing into appraisals of their characteristics in relation to traditional risk assessment schemes,^2^^,^^4–6^ the particular statistical modelling characteristics that would be necessary for their global use in various populations^7^ and their potential added public health value.^1^ In the case of coronary artery disease, recent studies consistently suggested lack of clinical utility when added to traditional clinical risk factors.^2^ However, for breast cancer screening and risk assessment, the situation might be somewhat different.^4^^,^^6^^,^^8^

Strong genetic predisposition to breast cancer due to rare high-risk functional mutations in susceptibility genes, such as *BRCA1* and *BRCA2*, are well-known and used for individual risk assessment. Apart from a few such known mutations, the genetic risk for breast cancer is highly polygenic, including various molecular pathways as for any common complex disease.^3^^,^^8^ The risk is also strongly affected by multiple environmental risk factors and—though often not explicitly considered—chance.^9^ Various clinical prediction models for individualized breast cancer risk have been developed, but their discriminatory power and calibration accuracy are still limited.^10^ Common genetic variation is one line of additional information that is believed to make headway in individual risk assessment.

Genome-wide association studies (GWASs) have identified close to 200 common genetic susceptibility variants that explain around 18% of the familial relative risk for breast cancer.^11^^,^^12^ Multiple studies using PRSs for breast cancer risk discrimination have derived the area under the receiver operating characteristic curve (AUC), with the highest values reached being around 0.7. Overall, the conclusions have been that including a PRS into traditional risk assessment models would improve risk predictions, at least statistically speaking.^10^^,^^12^ However, comprehensive calibration and integration of models incorporating classical risk factors and genetic risk to predict breast cancer risk have been lacking.

In this issue of the *International Journal of Epidemiology*, Chatterjee and co-workers present a coherent and extensive evaluation of calibration and discrimination of a risk prediction model, integrating a 313-variants PRS and traditional risk factors, in 15 European-ancestry cohorts from six countries (in total 239 340 women aged 19–75 years, with 7646 incident breast cancer cases).^13^ In a nutshell, addition of the PRS to clinical risk factors led to improved population-level risk stratification with good calibration, but the ability to predict breast cancer at an individual level remained poor (AUCs less than 0.7).

Chatterjee and co-workers emphasize the importance of evaluating absolute risks in order to determine the clinical utility of risk models.^7^^,^^13^ The authors have developed and distributed a software tool, iCARE, that allows the systematic building of absolute risk models by synthesizing information from different data sources. Environmental risk factors can fluctuate or have temporal trends, and therefore model calibrations are essential to produce unbiased estimates of risk for individuals with different risk factor profiles in each population. Whether the calibrated prediction models would be clinically relevant would then depend strongly on the clinical application under consideration. Models with modest discriminatory ability, such as those involving PRS for breast cancer,^3^^,^^4^^,^^6^^,^^10–13^ can identify a substantial fraction of the population at higher estimated risk than other individuals in the population. However, the majority of cases in a population can still arise outside the groups identified as being at high risk, unless the discriminatory ability of the underlying model is high.^3^^,^^13^^,^^14^

In fact, achieving high discrimination with normally distributed biomarkers, modestly associated with disease, for any common complex disease appears fundamentally implausible.^3^^,^^14^ The pivotal corollary for public health applications is, as elaborated by Rose in 1985 in his classic work, ‘a large number of people at a small risk may give rise to more cases of disease than the small number who are at a high risk’.^15^ This is exactly the case for breast cancer too, as concluded by Chatterjee and co-workers. They also commented, along the lines of Rose,^15^ that the overall situation calls for broader public health efforts to substantially reduce the population burden of breast cancer.^13^

In relation to their modelling, Chatterjee and co-workers projected that an improved PRS, achievable through larger GWASs, could lead to better risk stratification, i.e. in combination with other risk factors, it could achieve an AUC of slightly over 0.7. The authors emphasized that the iCARE models predict the risk of overall breast cancer, rather than specific subtypes. The risk models presented were also aimed at the general population and did not adequately capture the risk for women with strong family histories or carrying high-risk mutations. Thus, incorporation of subtype-specific risk predictions could result in improved identification of women who would benefit most from specific interventions. The potential additional role of epigenetic markers in the prediction models of breast cancer is also under active investigation, for example in relation to DNA methylation^16^ and micro-RNA markers.^17^

In addition, recent large-scale work has indicated that the breast cancer PRS strongly modifies breast cancer risk in the high-impact mutation carriers.^8^ Thus, the recently defined limited set of clinically most useful genes in sequencing analyses for protein-truncating variants and rare missense variants,^18^ might bring about new more comprehensive ways of assessing genetic risk for breast cancer in the general population. These kinds of combined approaches might lead to improved risk predictions as well as better cost-effectiveness and benefit-to-harm balance, for example via risk-stratified protocols in which those individuals with low risk would not be offered screening.^1^ Therefore, whereas the PRS approach on its own cannot provide high-enough sensitivity for screening programmes, the combination of rare familial risk and a PRS might provide useful guidance for population-wide approaches.

In relation to genetic risk and population health, we should also remain vigilant in public communication and keep in mind that the knowledge of risk, phenotypic or genetic, might not actually effectively change human behaviour.^19^ Thus, irrespective of great hopes and hype on individually predicted risk and ‘precision medicine’ in general, the challenge of societal promotion of a healthy lifestyle remains as important as ever.

## Funding

M.A.-K. is supported by a research grant from the Sigrid Juselius Foundation, Finland.

## Author Contributions

M.A.-K. conceived the idea. T.W. and M.A.-K. drafted and subsequently revised the text together. Both co-authors approved the final version. M.A.-K. acts as the guarantor for the work.

## Conflict of Interest

None declared.
